# Workplace mental health promotion in a large state organization: Perceived needs, expected effects, neglected side effects

**DOI:** 10.12688/openreseurope.13192.1

**Published:** 2021-03-24

**Authors:** Lilly Paulin Werk, Beate Muschalla

**Affiliations:** 1 Psychotherapy and Diagnostics, Technische Universität Braunschweig, Braunschweig, Lower Saxony, 38106, Germany

**Keywords:** workplace health promotion, prevention, qualitative study, needs, mental health, side effects

## Abstract

**Background:** Work ability and mental health in the workplace is increasingly promoted in terms of workplace health management. In order to select suitable interventions at work in a concrete context, employees and managers of a large state organization (science and development sector) were asked about perceived needs, desired effects and possible side effects of health promotion interventions.

**Methods:** 13 semi-structured interviews with managers and five focus group interviews with employees ( *N* = 20) were conducted in autumn 2020 by a behavior therapist in training. The evaluation was carried out by a qualitative content analysis of the interview transcripts according to a deductive procedure and was checked by two independent raters.

**Results:** Most frequently, need was expressed for individual case counselling by a health expert due to the diversity of work-related problems. Managers would like to see more health-related leadership training, and a review of the various communication channels of their organization. Expected positive effects are increased self-efficacy, higher person-job-fits and reduced absenteeism. Side effects were mentioned, such as confusion of health management activities with therapy, or sensitization effects when speaking too much about mental health in mentally healthy teams. Lack of competence with the topic of mental health was mentioned as a reason for non-participation in mental health promotion activities.

**Conclusions:** The role of managers in relation to mental health needs to be more defined. Side effects related to mental health activities should be considered in evaluations. Selection of health interventions should depend on the concrete needs of the organization.

## Introduction

As employees’ health is important for work ability and productivity, a variety of mental health-promoting training is conducted in the work context ( Chu *et al.*, 2000; Czabała *et al.*, 2011). According to the person-job-fit ( French, 1973) and the job-demands resources model ( Bakker & Demerouti, 2017), the workplace can have a positive impact on mental health and productivity if work demands fit well with the person’s capacities and the setting’s resources. The person-job-fit must be evaluated individually for each employee, because employees have different capacity profiles. However, do all employees need mental health promotion training? For employees who already have a good fit between demands and resources in their workplace, there might be no need for such training. This contradicts the “one size fits all” principle. Furthermore, thinking too much about one’s own stress can lead to sensitization effects and even have a negative impact on mental health ( Eriksen & Ursin, 2002).

For this reason, we conducted a qualitative investigation with employees and managers from a large state organization. We asked for employees and managers perceived needs for mental health promotion at work. Since little attention has been paid to side effects of work-oriented trainings ( Linden & Schermuly-Haupt, 2014), the interviewees were also asked about possible negative consequences of health-promoting trainings in the workplace.

### Mental health and mental health prevention at work

Mental health problems are frequent: about one third of the general population is affected by any mental health problem ( Wittchen *et al.*, 2011). Mental disorders since the early 2000s have been responsible for about twice as many incapacities to work in comparison to physical illnesses ( Linden & Weidner, 2005). An average of 5.7 cases of incapacity to work per 1.000 members were recorded by a national health insurance company due to mental health problems ( AOK, 2019). Under modern work demands, which increasingly require psychological capacities, persons with weak mental health often have problems in fulfilling achievements, or demands for endurance, flexibility or interactional capacities. Problems may occur in the form of work-related anxieties which often come with long absences, incapacity to work and disability ( Muschalla, 2016).

Longitudinal studies have shown that conditions at the workplace have a significant influence on mental health and work coping ( Chevalier & Kaluza, 2015). The German Association for Psychiatry, Psychotherapy and Psychosomatics (DGPPN) has addressed the interaction of work demands and mental health problems in a position paper that proposes preventive, curative and rehabilitative measures ( Berger *et al.*, 2012). Preventive measures include strengthening the resources of employees and organizing working conditions that fit to the employee’s capacities ( Berger *et al.*, 2012; French, 1973). In addition, mental health was included in the workplace risk assessment of many European countries ( Berger *et al.*, 2012).

In the treatment of work-related disorders and reintegration of employees on sick leave due to mental health issues, there is need to find the right person-job-fit ( French, 1973). Managers are often made responsible for assessing the demands of the workplace and finding the right person-job-fit, including for employees with health problems. This is only possible in cooperation with the employee’s physicians and company doctors. It was found empirically that employers have difficulties obtaining the relevant information for a stress assessment from employees ( Hofmann, 2014). Mental health organizations suggest addressing the prevention of the individual and actively counteracting burdens at the workplace that result from missing person-job-fit ( Berger *et al.*, 2012).

### Previous programs for workplace health promotion and their effects

A recent systematic review ( Bellón *et al.*, 2019) summarized findings from three randomized controlled trials (RCTs) from Finland, Japan and the USA. Only programs in group settings were found. As core interventions, stress management, social support, goal achievement and personal strengths were addressed to reduce mood disturbance symptoms.

Interventions used to increase mental health and well-being in the workplace are especially used for employees in the health system. Häggman-Laitila & Romppanen (2017) identified four studies about significant stress reducing interventions for nurse leaders: Three studies ( Pipe *et al.*, 2012; Tang *et al.*, 2010; Yong *et al.*, 2011) reported cognitive techniques and mindfulness to reduce stress, mood disturbances and anxiety. The fourth study used behavioral exercises to strengthen teamwork and communication ( Pipe *et al.*, 2012).

Lee *et al.* (2010) achieved significant positive effects in increasing emotional health and reducing burnout through leadership development training. Interventions have also been carried out to increase well-being in the workplace of general practitioners: four RCT studies - two with cognitive-behavioral techniques, one with mindfulness and one with health feedback and self-help information - all showed significant improvements in mental health ( Murray *et al.*, 2016). Studies on stress prevention among healthcare workers ( Ruotsalainen *et al.*, 2015) tested different preventive approaches within 14 RCT studies (e.g. cognitive-behavioral, participatory problem solving and decision-making, attitude change and communication). They found only short-term effects in stress reduction and no significant improvement over six months. Other review research found 10 RCT studies of preventive interventions for health care workers with positive effects on reduced job stress and higher job satisfaction ( Van Wyk & Pillay-Van Wyk, 2010). In this review only one study found medium-term positive effects on work demands and work satisfaction at 30-week follow-up ( Lökk & Arnetz, 2000). 

Interventions to increase mental health and well-being are also implemented in the education sector. Four studies used stress management training, school-wide coaching and mentoring support, among others ( Naghieh *et al.*, 2015). Overall, the effects on work ability, job-related anxiety and burnout were small ( Naghieh *et al.*, 2015). Programs for young adults in education refer in particular to positive psychology and mindfulness, with about half of the 57 studies in a systematic review ( Cilar *et al.*, 2020) showing significant effects for well-being, problem-solving and stress reduction.

### Side effects of mental health interventions

An aspect that has been somewhat neglected until now is that any kind of intervention may have not only positive impacts, but also negative side effects. Only recently, side effects have become a seriously discussed and investigated phenomenon in the field of psychological intervention research ( Linden *et al.*, 2020; Schermuly & Graßmann, 2019). Mental health prevention intervention in organizations can have several negative side effects, such as dysfunctional sensitization of employees and managers on mental wellbeing, e.g. focusing too much on potential harms, wellbeing, and a misunderstanding of mental illness as being caused by the workplace (“I believe work has made me sick”). As another problem, unrealistic expectations may occur such as “Work must make me happy every day and if it does not, it is not the right job for me”. Such unrealistic expectations may give rise to problems that would not have come up if “wellbeing” had not been induced as a major topic within a mental health campaign.

For example, frustration was reported after leadership workshops, as the implementation of the training content into everyday work was not successful because the content was too theoretical and there was a lack of support for implementation by supervisors reported ( Lee *et al.*, 2010). There is a lack of evaluation of long-term effects of various mental health training programs, and it has been suggested that workplace interventions should address more specific stressors ( Ruotsalainen *et al.*, 2015). Despite short-term effects for reduced stress and improvement of job satisfaction, it may be that there is no evidence of reduced staff absenteeism ( Van Wyk & Pillay-Van Wyk, 2010). It was even found that there were no significant effects of workplace interventions on the long-term return to work process ( Van Vilsteren *et al.*, 2015).

Some studies show strong drop-outs or non-participation ( Murray *et al.*, 2016): from an original sample of 338 students in an online program, 234 dropped out because they made a conscious decision not to continue participation, or teachers withdrew students from the program ( Burckhardt *et al.*, 2015). Another program had a non-response rate of 32.6% ( Weinberg & Creed, 2000): reasons for non-response were the additional time required for participation in the study, and the fact that support staff and doctors in particular did not feel addressed by the topic of mental stress. A comparison of drop-outs from control and intervention groups found that all drop-outs were “healthier” participants who did not feel the need to participate ( Gardiner *et al.*, 2004). Also, interventions are particularly used by those who do not urgently need them (“preach to the converted”, Holt & Del Mar, 2006).

Furthermore, interventions can have negative effects if qualified professionals with knowledge about skills and mental health promotion do not take over the management of such interventions ( Cilar *et al.*, 2020).

In sum, studies until now have shown the short-term effectiveness of different workplace interventions on stress levels, job satisfaction, depressions symptoms, team climate and well-being. However, it is until now unclear which interventions are suitable for whom and which interventions also have long-term effects on work ability, RTW and absenteeism. Furthermore, drop-out and non-participation have been identified as problems, which leads to the question of how much workplace mental health initiatives are senseful and for whom.

In order to fill this research gap, this present study has been conducted as the first part of a longitudinal project that covers needs analysis, interventions and their evaluation in different organizations in Europe (H-WORK, De Angelis *et al.*, 2020). The relevance of mental health in the workplace as perceived by managers and employees, and needs for health promotion interventions are investigated qualitatively.

### Research question

This study investigates attitudes and needs in relation to mental health in the workplace as perceived by managers and employees. In terms of the legally defined risk assessment ( Bundesrat, 2013; WHO, 2008), information is to be gained on how this is implemented in public institutions and organizations and what support managers and employees still need and do not need within the workplace for health promotion.

Research questions are: 

1.   What are the **current unsolved problems** regarding mental health at work as perceived by managers and employees of a public institution?

2.   What can be done to solve these problems and **which positive effects should result from suggested activities and interventions**?

3.   What **problems and side effects** may be associated with the implementation of these proposed interventions?

## Methods

The conduct of the study was reviewed and approved by Horizon 2020 and the ethics committee of the Faculty of Life Sciences at the Technische Universität Braunschweig, (ethics approval number D-2020-07). Written informed consent for participation and publication of the participants’ anonymized data was obtained from the participants.

### Setting

This study was carried out in a large state organization in Northern Germany, which belongs to the sciences and development sector and is self-administrated. The organization consists of six overall (administrative) departments with subunits and four research and development centres. In sum, 6418 people are employed in the organization, i.e., 243 managers and 4678 subordinate employees in research and development, and 1497 managers, employees and trainees work in administration.

### Participants

All 6488 employees of the organization were informed about the project by email and got the invitation for participation in needs analysis interviews. Two managers reported back willing to participate without being contacted personally. 15 managers, 22 employees and one security officer were in a next step invited by personal contact: from each of the organization’s six departments, on average three managers were asked to participate, and asked to name employees from his/her unit who were willing to participate as well.

In sum, 34 of the 40 personally invited reported back with interest to participate. Semi-structured interviews have thus been done with 13 managers and 20 employees. The average age of the managers was *M* = 45.38 years (range: 32–65 years) and five out of 13 were men. Five managers belonged to the research and administration staff and eight to the administration staff. The average age of the employees was *M* = 40.1 years (range: 19–63 years) and two out of 20 were men. Seven employees belonged to the research and administration staff and 13 to the administration staff.

The needs analysis interview was carried out through semi-structured interviews according to guidelines and lasted about one hour. The interview was done by a Master psychologist (L.W.) who was presently in training as a behavior therapist, and thus well trained in interview techniques and structured exploration. The qualitative survey was done in September-November 2020. Sound recordings and word-by-word transcripts were prepared for those interviews for which consent was given. The transcripts were not corrected by the participants, the interviewer took additional notes. The interviews were analyzed through a qualitative content analysis using a deductive approach. With the help of the MAXQDA 2020 software (a freely available alternative software is QDA Miner Lite), categories were built and the texts from the interviews were coded according to the research questions. The coding tree is available as *Extended data* ( Werk & Muschalla, 2021). Cross-tabulations were calculated with kappa statistics. An inter-rater reliability of κ = .63 was determined with a code overlap of 70% between two independent trained raters. Participants were offered the option to receive a summary of the study´s results after study completion.

### Semi-structured interview

Interviews were conducted by a female trained Master psychologist (L. W.) who was in training to become a behavior therapist. The participants knew the aim of the study, the professional background of the interviewer and were informed about the topics in advance. Before the interview, the interviewer and the participants were unknown to each other. In the first step, the interview partners were asked for basic socio-demographic data (age, position, department, size of the team, length of employment in the organization). Using a structured interview guide, they were then asked about problems regarding mental health and well-being that occur in everyday working life. Subcategories such as communication, stigmatization, leadership, demands and COVID-19 were mentioned. Employees and managers were asked to describe ideas for solving problems and desired positive effects. They could refer to resources in the workplace and previous interventions. Concrete intervention contents, settings and designs were discussed. In the last step, the interviewer asked for possible barriers, problems and disruptive factors in the implementation of such activities and interventions. Participants were able to draw on previous experiences or freely consider what problems and side effects they could imagine. 12 manager interviews and three focus groups were conducted face-to-face, one manager interview and two focus groups took place online. The face-to-face interviews were conducted in the offices in the workplace without any other persons present.

### Non-participants and drop-outs

Six participants were asked for an interview and decided not to participate. One participant withdrew from the interview after reading the consent form. The reasons are shown in Table 1.

**Table 1.** Reasons for non-participation in the qualitative interview on needs for mental health promotion in the workplace.

Non- participants	Role in organization	Reason for non-participation
**1**	Manager	He said there was no need for mental health interventions, this is not a priority at his workplace.
**2**	Employee	He has no relation to mental health.
**3**	Manager	Due to COVID-19 there is a lack of time for the interview participation.
**4**	Manager	Due to COVID-19 there is a lack of time for the interview participation.
**5**	Security Officer	She reported work overload, did not want to take part in the interview in order to take care of her own mental health.
**6**	Manager	His team would be too small for him to make a statement about mental health as a manager.
**Drop-outs**	**Role in** **organization**	**Reason for drop-out**
**1**	Employee	After reading the consent form, he decided not to participate, as data collected is too personal.

## Results

### Current unsolved problems

Both employees and managers mentioned that employees need a mental health expert (who is not member of their own working team) to discuss individual work-related problems. This problem was mentioned 29 times ( Table 2). 10 out of 13 managers mentioned the problem of insufficient leadership training on healthy leadership and dealing with mental health in the workplace. This problem was not mentioned by employees. Most managers (10 out of 13) said that there were structural communication problems within the organization, with information being spread over too many different channels or being too unstructured. Lack of communication between the organization’s management and the departments, as well as inconsistent transmission of facts were mentioned by managers less often. Only one manager out of 13 criticized the reintegration management’s support in cases of employees with a long period of sick leave. Managers focused on their role as leaders, while employees’ interest rather touched structural problems (e.g. further training opportunities). Managers and employees agreed that there was a need for individual counselling for work problems instead of a broad range of unspecific general information or seminars.

**Table 2.** Reported problems and suggested solutions for mental health issues in a large organization (research and development and administration) given by managers and employees in a semi-structured interview ( *N* = 33).

Role	What is the present (unsolved) problem? (x) ^1^	What has already been done? Which interventions are existing in the organization?	What should be done? IT Information & Training D Specific Diagnostics P Preventive Intervention C Curative Interventions R Rehabilitative Interventions S Structural changes in organization	Why should this be done? Which positive effect is expected?	Which problems may occur? Which side effects are expected from the activities that should be done?
**Manager** **( *n*= 13)**	Lack of sufficient counselling services for individual work problems (11)	Counselling in the field of reintegration management and addiction counselling	DPC: low-threshold individual counselling on work-related problems by qualified personnel without long waiting times	Strengthening self-efficacy, behaviour-oriented problem solving	Counselling may be perceived as therapy, there may be too high expectations, negative reactions of the environment towards behavior changes of the coachee
	Managers are not trained (enough) for mental health issues (10)		I: Inform managers about mental health at work, guidelines on competent, mentally healthy leadership I: Make the boundaries clear: managers are not doctors. Managers cannot be made responsible for their employee’s health status		Managers are not trained (enough) for mental health (10x)
	Information is scattered unstructured through different channels (10)	Website, information portal, various newsletters sorted by topics	I: Reorganization of information channels, one central channel for work health issues	Easier search for specific information	Lack of information during restructuring, technical overload, getting used to the new channel
	Previous (preventive) interventions by occupational health management were not attended (6)	Mental health-related seminars are designated as low-threshold as possible (“Training for solving unsolvable problems”)	I: Naming and rewriting “psychological interventions” differently, e.g. in the sense of “mental fitness” training, or “work- related counselling”. I: Making a new selection of which interventions are helpful and necessary and which are not	Increased registrations for “psychological interventions”	No need for preventive offers, fear of stigmatisation when signing up in a psychological intervention
	Low cohesion in teams (4)		IT: Seminars on team cohesion, conflict solution, communication, problem-solving strategies		Remaining low cohesion in teams
	Too much bureaucracy in decision-making (3)	Responsibilities for various bureaucratic matters communicated within the university: Who is responsible for which topic?	S: Reduce bureaucratic decisions at state level, clearly separate administration from science	More time for content work, lower workload	Lack of feasibility due to laws (e.g. GDPR)
	Work overload of employees (3)		PC: Voluntary working time recording, questionnaires for self-evaluation of one's own work behavior, seminars on work-life balance and warning signs of mental overload		
	Role of middle management not clearly defined (3)		I: Guidelines for the manager's own area of responsibility: Who is responsible for what (and for what not) in the field of occupational health?		
	Neglect of person-job-fit when tasks are delegated or reorganized (2)	Job descriptions are available, employee appraisals are offered	I: Managers are informed about the importance of person-job-fit D: Employee interviews, feed-back discussions: Does your workplace fit your qualification, capacities and abilities?	Higher job satisfaction, less work overload and work underload	Lack of personnel, employees do not dare to address missing person-job fit
	Facts are presented differently at different organization levels or are not disclosed (2)	Clear or flat hierarchies, various communication channels	I: Seminars on communication of information S: greater networking between departments	Fewer conflicts, improved cooperation, better work results, greater “we” feeling of the organization members	
	Organization’s management has hardly any contact with departments (2)	Not known	PC: Talks between organization’s management and individual managers and employees	Identification with the organization, improved communication of all levels	Communication with organization management creates pressure, sense of supervision
	Lack of support after long incapacity to work (1)	Reintegration interviews	D: Check the fit between job- related capacities and job demands R: Workplace with degrees of freedom/tolerances for failures	Reducing periods of sick leave, easier return to work	Sensitization effects, lack of knowledge of reintegration management about matching capacities and jobs
**Employee** **( *n*= 20)**	Lack of sufficient counselling services for individual work problems (18)	Counselling in the field of reintegration management and addiction counselling	DPC: Low-threshold individual counselling on work-related problems by qualified personnel without long waiting times	Strengthening self-efficacy, behaviour-oriented problem solving	Counselling may be perceived as therapy, there may be too high expectations, negative reactions of the environment towards behavior changes of the coachee
	Lack of professional competence of administrative staff (e.g. speaking to relatives of deceased persons) (3)		I: Guidelines for dealing with specific workplace problems		
	Lack of identification with the university and its mission statement (2)	Speeches by the university management, mission statement on the website, events for all staff		Higher job satisfaction, motivation, cohesion	Too much identification could lead to overwork
	No possibility for further qualification in administration (1)		I: Communicate the natural limits of career possibilities in administration jobs right on recruitment (“realistic job preview”)		

^1^Number of times the problem was mentioned

### Expected positive effects

Managers and employees hoped for increased employee self-efficacy and problem-solving skills by means of counselling for individual problems (29 out of 33). Organizational changes, such as the restructuring of information flows, reduction of bureaucracy, or identification with the organization and its mission statement, are intended to make information and interventions more accessible, reduce workload and improve cohesion (15 out of 33). Overarching expected positive effects relate to higher job satisfaction, less work overload or underload, higher person-job-fit, and reduced absenteeism ( Table 2).

### Possible side effects

A variety of possible side effects were mentioned within the semi-structured interviews: the counselling may be perceived as “therapy”, or may raise expectations too high, although this could be avoided with only a few sessions and a focused goal (e.g. goal: “learning to work with weekly to do lists in a senseful way”, but not: “healing a mood disorder”, which the coachee may also have as a general health problem outside of work) (3 out of 33). The social environment of the participants - family, friends or colleagues - could react negatively, if the behavior of the coachee changes (“Why doesn’t she want to do this task? She has always carried out this task until now”) (5 out of 33).

During restructuring of the information channels, information may be lost or new versions of programs could be technically overwhelming and therefore not used (3 out of 33).

As the needs analysis showed that no further preventive interventions are needed in a large organization, preventive interventions might even be in danger of causing sensitization effects (“I never thought about this before, but maybe my workplace makes me sick?”), or participants may be afraid of stigmatization (“This colleague went to the work coaching, I believe he has a mental health problem”) (5 out of 33).

Reducing bureaucracy could fail in its implementation because laws cannot be circumvented (e.g. GDPR) (8 out of 33).

In order to create a better person-job-fit, employees must communicate unbalanced demands and resources to managers and there must be enough staff, which is not the case everywhere (4 out of 33). The reintegration management has a lack of knowledge about matching capacities and jobs, in case there is no physician who can judge whether a health-related problem causes need for a specifically adjusted workplace. Managers are not doctors and cannot answer the question of whether an employee has work-related problems due to an illness (4 out of 33).

More communication with organization management could create pressure for the employees and too much identification with the organization could lead to overwork (2 out of 33).

The reasons for non-participation may also add to this point: reasons for non-participation were perceived overload with the topic, having no time or not feeling competent enough to deal with the mental health of employees ( *N* = 6).

## Discussion

### Unsolved problems and expected positive effects

Our findings show that both employees and managers who were interested in the topic of mental health at work mentioned the ***need for individual counselling and problem-solving***. They suggested that such activities should be done when a concrete situation with the need for counselling arises, but not “preventively”. Thus, individual counselling for work problems should be done instead of general global mental health information and actions. In the literature, this aspect has been discussed as well ( McLeod, 2010; Rongen *et al.*, 2013) by means of individual interventions at work such as motivational interview-based health coaching ( Butterworth *et al.*, 2006), stress counselling in the workplace ( Cooper & Sadri, 1991), counselling programs for alcohol-related problems ( Guppy & Marsden, 1997) or individual workplace training ( Oakman *et al.*, 2018). They are flexibly adjustable to employees’ skills and job requirements ( Grant, 2005), individual stress and psychological symptoms ( McLeod, 2010) and work-related issues and problems ( Hughes & Kinder, 2007). 

****Information**** is also appreciated, but information must be adjusted to the needs of the recipients: e.g. managers need information about what to do when an employee is on long term sick leave, employees may need information on services that can be consulted in case of problems at work. In our investigation of a large organization with several departments and subunits here, information dissemination and information overkill were mentioned as problems. This is a structural problem that has also been reported in empirical research before: work effectiveness of health workers can be impaired by information overload in the clinical environment ( Hall & Walton, 2004). In healthcare services ( Wilson, 2001) and among emergency managers ( Misra *et al.*, 2020), the overload of information leads to higher stress levels. In the university context, there is an immense overload of digital information and this can have a negative impact on mental stress (“technostress”) and self-management capacities ( Misra & Stokols, 2012).

### Side effects and critiques

As a critique, it was suggested that managers have ****limited competency**** for dealing with mental health. They are not doctors and should not be made responsible for employees’ health status. Mental disorders are by their nature not caused by work conditions, but work conditions can be more or less appropriate for different people with different capacities and health statuses ( Muschalla, 2016).

Some managers pointed out the possibility of ****side effects**** that may occur as a consequence of information or requirements: counseling may be misunderstood as a kind of “therapy“; responsibilities for creating a person-job-fit for one’s employees may be rejected due to the idea that mental health is purely a topic for physicians; and mental disorders may be confused with mental demands or healthy stress reactions (which may normally occur due to intensive work phases even if they are followed by routine work).

Side effects and possible barriers and problems in implementation that were mentioned by the interviewed employees here can be compared with other findings: extant literature suggests that managers might refuse to take responsibility for employees’ mental health status or mental work ability due to own problems with mental health or work overload ( Martin *et al.*, 2018). In addition, it can be difficult to strike a balance between respecting the employee’s privacy and sufficient problem exploration ( Ladegaard *et al.*, 2017). In interviews, managers reported “cross pressure” between content work and leadership behavior, too little support from the organization and a lack of systematic risk assessment, which is why they would avoid addressing mental health ( Ladegaard *et al.*, 2017).

In many studies on mental health interventions, side effects and negative effects have not been assessed explicitly: Murray *et al.* (2016) looked at whether drop-outs were reported, but side effects are not addressed in their systematic review. Often, non-participation and drop-outs are not reported at all (e.g. Tan *et al.*, 2014). Side effects are until now not mentioned systematically despite the fact that they can have a major impact on the effects of interventions. They should be communicated to the participants and taken into account in the evaluation ( Linden & Schermuly-Haupt, 2014; Schermuly & Graßmann, 2019). Side effects of mental health promotion in organizations have until now been underrecognized ( Cilar *et al.*, 2020) and are in need of further research.

## Conclusions

This article presents the current unsolved problems related to mental health promotion in the workplace from the perspective of employees and managers of a public institution. Workers were asked which interventions would meet their needs and what positive effects they would have, as well as which interventions they do not need. The interviews showed that employees and managers express similar needs; in particular, individual counselling and a regulation of information channels were considered most helpful by both parties. The role of managers in maintaining the mental health of employees in the workplace needs to be more clearly defined so that managers are not held responsible for the health status of their employees. Side effects, like managers’ rejection of person-job fit or sensitization effects, were discussed in the context of work-related training. Further research is needed into employee work ability on an individual level, based on the person-job-fit model. In the evaluation of individual workplace training, side effects should be collected in order to better assess cost-benefit ratios. The question is “Which mental health (information) interventions fit for whom and who needs what type of support at work (if any) in order to do a good job?”

## Data availability

### Underlying data

The raw data collected come from interview transcripts and are subject to a high level of confidentiality and security despite anonymization. Due to this, data are reported in aggregated form in Table 2 to protect individual participants. The data are kept at the Institute of Psychology, Technische Universität Braunschweig on its own protected server and can be requested in justified cases (e.g. colleagues who want to undertake a comparative study, or a review) via the authors’ e-mail addresses ( b.muschalla@tu-braunschweig.de and l.werk@tu-braunschweig.de). 

### Extended data

Open Science Framework: Workplace mental health promotion in a large state organization: Perceived needs, expected effects, neglected side effects: Data and Qualitative Coding Tree. https://doi.org/10.17605/OSF.IO/2A3ZP ( Werk & Muschalla, 2021).

This project contains the following extended data:

- Aggregated data from the qualitative interviews, and the coding tree in PDF format

Data are available under the terms of the Creative Commons Attribution 4.0 International license (CC-BY 4.0).
