# Supplementary material for: Workplace mental health promotion in a large state organization: Perceived needs, expected effects, neglected side effects
Source: Open Res Eur. 2024 Oct 15;1:17. Originally published 2021 Mar 24. [Version 2] doi: 10.12688/openreseurope.13192.2 (PMC11562386; doi:10.12688/openreseurope.13192.2)
Supplement: Peer review history

# Reviewer report: Reviewer response for version 2

**DOI:** 10.21956/openreseurope.20028.r47243

**Published:** 2024-12-20

**Authors:** Sabine Rehmer

**Affiliations:** 1 SRH Hochschule für Gesundheit, Gera, Germany

The study itself is interesting and, with its qualitative research approach, provides a good starting point for generating hypotheses in relation to the research questions. First of all, I would like to thank the authors for their work. In addition to the many positive aspects, I believe that some aspects should be revised. These are presented below. I wish you all the best with the revision!

**Structure and literature:**

 The structure of the work is clear and precise. However, the cited literature is often not very up to date, which should definitely be revised! Information on the prevalence of absenteeism and early retirement is also outdated. It should also be noted that the data from the AOK, for example, are given, but other health insurance companies also provide statistics on this.

**Study design and academic value of the work:**

 When presenting the sample, it is not clear to what extent gender has a cross-effect with the organizational unit – please provide the assignments to gender and organizational unit in a cross-tabulation for managers and employees.

 When creating the interview guide, no information on quality management is presented, for example, cognitive debriefings with representatives of the target samples or experts and, if necessary, revision of the questions. It seems unusual that the analysis was carried out purely according to a deductive approach, according to the method description. Particularly in the case of semi-structured interviews, it seems implausible that no content was mentioned that was inductively incorporated into the data analysis.

 Detailed information is missing for the focus group interviews conducted with employees.

 The inter-rater reliability of κ = .63 is only moderate for scientific studies and, in my opinion, insufficient.

 In the presentation of the results, it is not clear how many statements the further results in columns 3-6 refer to. In addition to some statements that were mentioned by many people, many statements are also presented that refer to the mention of very few people. The presentation distorts the results.

**Conclusions:**

 The results can only be partially traced back to a few statements, so that a generalization should be presented in a much more limited way. Also, as already mentioned, there is no information on how many statements the additional information given on the problem refers to. This should definitely be added.

Is the study design appropriate and does the work have academic merit?

Partly

Is the work clearly and accurately presented and does it cite the current literature?

Partly

If applicable, is the statistical analysis and its interpretation appropriate?

Partly

Are all the source data underlying the results available to ensure full reproducibility?

No source data required

Are the conclusions drawn adequately supported by the results?

Partly

Are sufficient details of methods and analysis provided to allow replication by others?

No

Reviewer Expertise:

occupational health and safety psychology

I confirm that I have read this submission and believe that I have an appropriate level of expertise to confirm that it is of an acceptable scientific standard, however I have significant reservations, as outlined above.

# Reviewer report: Reviewer response for version 2

**DOI:** 10.21956/openreseurope.20028.r45554

**Published:** 2024-11-08

**Authors:** Ina Kayser

**Affiliations:** 1 IST University of Applied Sciences, Duesseldorf, Germany

This study is a thoughtful and well-constructed examination of workplace mental health interventions, particularly in a public sector context. It challenges the idea of one-size-fits-all interventions and advocates for a more tailored approach. Despite minor areas for improvement in terms of structure and detail, the study offers valuable insights for both researchers and practitioners interested in occupational health and organizational policy.

 Major Comments

1. The study makes an excellent case for why workplace mental health interventions need to be personalized. However, a more explicit emphasis on the unique setting—a public institution in Northern Germany—would enhance the reader’s understanding of why this study stands out. Public organizations, as opposed to private enterprises or SMEs, often face distinct challenges, so highlighting this context would reinforce the study’s significance. Additionally, it would be helpful to position the study more clearly within the existing gap in qualitative research on workplace mental health.
2. While the literature review is comprehensive, it could benefit from a more focused synthesis to improve readability. Grouping studies by intervention types (e.g., cognitive-behavioral, mindfulness) or outcomes (e.g., stress reduction, job satisfaction) would allow readers to better identify key patterns in previous research. This would also make it easier to understand how the current study’s focus on individual needs and the potential downsides of generalized interventions contribute new insights.
3. The study uses the Person-Job Fit and Job Demands-Resources models as theoretical foundations, but these models could be more fully integrated into the discussion of results. Explicitly connecting the study’s findings to these models in the Discussion section would help clarify how the results contribute to or expand upon these frameworks, particularly in the context of real-world application within large organizations. This would deepen the theoretical value of the study.
4. The qualitative approach is appropriate for exploring employee and manager perspectives. However, the choice to recruit participants through the occupational health manager may introduce selection bias. It would strengthen the methodology section to acknowledge this more explicitly and discuss how it might affect the findings. Providing a more detailed rationale for why only certain departments were included in the interviews would also enhance transparency and help readers better understand how representative the sample is.
5. The Conclusion effectively summarizes the need for more individualized approaches to workplace mental health. Adding a few practical recommendations, such as encouraging modular mental health programs where employees can select relevant components, would make the findings more actionable for decision-makers. This would allow organizations to see clearer pathways to implement the study’s recommendations.

Minor Comments

1. The literature review could briefly mention how the study’s approach differs from existing qualitative research, especially if specific qualitative studies have directly influenced this work. This would underline the study’s originality in addressing gaps that have been less explored.
2. While the Results section is thorough, it could be organized around key themes (e.g., “Unsolved Problems,” “Expected Benefits,” “Side Effects”) to make it easier to follow. A clearer structure within the results would help readers quickly identify which findings correspond to which research question, improving the section’s overall readability.
3. The qualitative content analysis is well-described, but including more information on why content analysis was chosen over other qualitative methods would strengthen the Methodology section. Additionally, explaining how codes were derived or providing a sample coding tree could make the analysis process more transparent.
4. While the study acknowledges its limitations, mentioning additional ones, such as potential subjectivity in qualitative coding or reliance on self-reported perceptions, would balance the Limitations section. These points would provide a fuller picture of the findings’ scope and applicability.
5. The discussion of potential side effects, like sensitization effects or stigmatization, is insightful. Adding a few practical examples of how organizations can address or mitigate these risks would make this discussion more tangible. For instance, brief recommendations on training managers to handle these issues or guidelines on setting realistic expectations for employees would be useful.
6. The Discussion could be slightly more streamlined by integrating comparisons with previous research throughout, rather than waiting until the end. This would make it easier for readers to see where findings align or contrast with existing studies and keep the flow of ideas more cohesive.

Is the study design appropriate and does the work have academic merit?

Yes

Is the work clearly and accurately presented and does it cite the current literature?

Partly

If applicable, is the statistical analysis and its interpretation appropriate?

Not applicable

Are all the source data underlying the results available to ensure full reproducibility?

Yes

Are the conclusions drawn adequately supported by the results?

Yes

Are sufficient details of methods and analysis provided to allow replication by others?

Partly

Reviewer Expertise:

Inforamtion Systems, Employee well-being through IS, Acceptance, IS and health

I confirm that I have read this submission and believe that I have an appropriate level of expertise to confirm that it is of an acceptable scientific standard, however I have significant reservations, as outlined above.

# Reviewer report: Reviewer response for version 2

**DOI:** 10.21956/openreseurope.20028.r45191

**Published:** 2024-10-24

**Authors:** Irene Jensen

**Affiliations:** 1 Karolinska Institutet, Solna, Sweden

This study aims at investigating employees and managers’ views on work place mental health promotion. The topic is of great importance. I have, however, noted several queries that needs to be addressed.  Below I have commented on small and more severe problems with this manuscript.  

*Introduction*

  I suggest that the authors rephrase the last sentence in the first paragraph “thinking to much about one’s own stress can lead to sensitization effects………. The authors simplify a very complex phenomena and the reference given is quite old.

 Overall, the references in the introduction and background are not the most recent ones -the manuscript would benefit from updating the references.

 In the mental health section third line from the bottom first para “psychological capacities” please define what this concept refers to – do the authors mean cognitive ergonomics?

 Further please define what type/diagnosis the concept mental health issues refer to.  It is important in the context of workplace health promotion and from the perspective of workplace conditions affecting mental health. Overall, all used concepts including workplace health promotion need to be clearly defined in relation to this study.

 References are sometimes addressing students and health promotion efforts. However, the students study environment is of importance, but it cannot be directly translated to workplaces for adults in private or public enterprises.

*Method*

 About the sample – no inclusion criteria other than managers or employee is stated. In the interpretation of the results, it would help to know if the participants themselves have had - or suffers now  from mental health problems. Since the sampling method used is self-selection it might be a biased selection with people with negative personal experiences of - not promotional activities but more of rehabilitative interventions.

 It is nice to have the reasons for dropout included.

 The manuscript would benefit from a more detailed report on the method including the interview guide and the analysis

*Results*

 Fifth line first para the table ref is not correct I presume it should be table 3

 As pointed out by another reviewer, the result of this qualitative study is presented in counts which is unusual for qualitative studies since the sample is not representative and no generalization to larger cohorts can be made. Please clarify and describe more about the analysis approach.

*Side effects and critiques*

 First para second line “Mental disorders are by their nature not caused by work conditions but work conditions can be more or less appropriate for different people with different capacities and health statuses “. This statement is not correct if you consider the vast research literature showing that work can cause mental illness – most commonly such as burn-out, depression, anxiety. As mentioned above, the authors must state clearly what they are studying by defining the concept mental health and illness as now it is too vague -the statement above is only correct when relating to psychiatric morbidity such as f ex personality disorders, schizophrenia etc.

*Limitations*

 The author states that the results can be interpreted as from a prototypical sample. They state this on their assumption that the recruitment might have attracted mostly employees who have already been in contact with the occupational health manager but, and this is important, the authors do not know. As mentioned above the inclusion criteria should have included statements on whether they wanted people with personal experience of mental illness or not and applied in the sampling process.

*Conclusions*

 Overall, it is unclear throughout the manuscript if they are studying and reporting on promotion or rehabilitative efforts or both, and what type of mental health and illness the study aims at. Thus, the conclusions made are not useful because of this confusion.

Is the study design appropriate and does the work have academic merit?

Partly

Is the work clearly and accurately presented and does it cite the current literature?

Partly

If applicable, is the statistical analysis and its interpretation appropriate?

Not applicable

Are all the source data underlying the results available to ensure full reproducibility?

Yes

Are the conclusions drawn adequately supported by the results?

No

Are sufficient details of methods and analysis provided to allow replication by others?

No

Reviewer Expertise:

Occupational health, worker health

I confirm that I have read this submission and believe that I have an appropriate level of expertise to state that I do not consider it to be of an acceptable scientific standard, for reasons outlined above.

# Reviewer report: Reviewer response for version 2

**DOI:** 10.21956/openreseurope.20028.r45167

**Published:** 2024-10-22

**Authors:** Michael Donnelly

**Affiliations:** 1 Centre for Public Health, Queen's University Belfast, Belfast, UK

The revised version of the paper reads much more fluently and it is more informative about workplace mental health promotion. The revisions are limited necessarily because, as the authors note in their response, the study was conducted and concluded several years ago.

Is the study design appropriate and does the work have academic merit?

Partly

Is the work clearly and accurately presented and does it cite the current literature?

Partly

If applicable, is the statistical analysis and its interpretation appropriate?

Not applicable

Are all the source data underlying the results available to ensure full reproducibility?

Partly

Are the conclusions drawn adequately supported by the results?

Partly

Are sufficient details of methods and analysis provided to allow replication by others?

Partly

Reviewer Expertise:

Public health and health services research including the evaluation of mental health care interventions and services.

I confirm that I have read this submission and believe that I have an appropriate level of expertise to confirm that it is of an acceptable scientific standard.

# Reviewer report: Reviewer response for version 1

**DOI:** 10.21956/openreseurope.14262.r37881

**Published:** 2024-02-20

**Authors:** Charlotte E Hall

**Affiliations:** 1 Department of Psychological Medicine,, King's College London, London, England, UK

Thank you for the opportunity to review this paper which focuses on workplace mental health promotion in a large state organisation. The paper is an interesting read, but I believe some additional work/adjustments could be made to the manuscript to make it more comprehensive. I hope you find this feedback helpful; my suggestions are made below.

- The majority of the studies in the introduction seem to be quantitative or review based. For the purpose of an introduction for a qualitative study, it would be useful for the reader to know what qualitative research findings/headlines are prevalent in the literature on this topic.
- A reference to a broader longitudinal study (H-WORK) is mentioned at the end of the introduction, it may be useful to expand of what this project aims to do and is looking to achieve to better situate the methods.

- More information is needed on the recruitment of participants. For example, how were the 15 managers, 22 employees and one security officers chosen to be personally contacted? Were these amounts indicative of staff percentages or just chosen at random(?) and how were the three managers from each of the six departments chosen to be contacted?
- “Five managers belonged to the research and administration staff and eight to the administration staff.” Please could more detail be added so each of the staff departments are known as well as how many managers/employees in each. It would be useful to know which ones you were unable to get participants from.
- The table of reasons for non-participation is very useful. Another table detailing the participants ‘basic socio-demographic data’ that was collected at the start of the interviews would be good.
- Guidelines are mentioned with no supporting reference, please expand details or add reference.
- “Sound recordings and word-by-word transcripts were prepared for those interviews for which consent was given.” Were transcripts typed verbatim by the researcher, or was software used for transcription?
- Please could you justify in the text why it was chosen to use a content analysis methodology on interview and focus group data when it is more commonly chosen to use a thematic based approach and derive themes from saturated data? With this in mind, did you reach data saturation, or were new themes still emerging from the interviews/focus group data?
- The side effects mentioned in the results/discussion are very interesting.
- Additional things to note, there is variation in some of the numbers reported, please check for consistency (e.g., 6418 vs 6488 employees).

Is the study design appropriate and does the work have academic merit?

Partly

Is the work clearly and accurately presented and does it cite the current literature?

Partly

If applicable, is the statistical analysis and its interpretation appropriate?

Not applicable

Are all the source data underlying the results available to ensure full reproducibility?

Partly

Are the conclusions drawn adequately supported by the results?

Partly

Are sufficient details of methods and analysis provided to allow replication by others?

Partly

Reviewer Expertise:

Occupational mental health

I confirm that I have read this submission and believe that I have an appropriate level of expertise to confirm that it is of an acceptable scientific standard, however I have significant reservations, as outlined above.

# Response

**Published:** 2024-09-03

**Authors:** Beate Muschalla

**Affiliations:** Technische Universität Braunschweig, Germany

Thank you for this comment. We have added a section on previous qualitative research in the field of workplace health promotion to the theory section (line 129-162). We have provided an explanation of how the needs analysis is embedded in the project context: “The first project step is the needs analysis, the results of which are reported here, aims to explore the needs for interventions to promote mental health in the workplace from the perspective of employees and managers on the basis of interview data and evaluation using qualitative content analysis. The needs analysis is followed by the implementation of the derived interventions in the workplace, their conduction and evaluation based on several measurement points.” (line 209-214). Your question about the recruitment of participants addresses an important limitation of the study. In the first recruitment step, all 6418 employees were contacted via a mailing list. As the response rate was poor (only two managers responded), it was necessary to recruit personally. This was done by the occupational health manager, who is very well networked within the organization and approached people from her sphere of influence. Random recruitment was not possible due to the size of the organization. We have now specified this in the method and highlighted it as a limitation in the discussion (line 256-261). We have added a more precise description of the organizational structure in the method to better illustrate which sample we have reached (line 263-274). The request for the basic socio-demographic data was a good idea and we have included another table displaying this (Table 1). We have added references for the interview guide and the following sentence: “The protocols were typed up word for word by the researcher and assistants.” (l. 286-287) Regarding your question of the use of a content analysis methodlogy, this is partly for methodological reasons, and partly because qualitative content analysis is better suited to the objectives of our project. On the one hand, the interviews were all conducted before the evaluation, so that no saturation point could be determined. Secondly, it was a major challenge in our approach to find enough participants in the first place and to conduct interviews and focus groups with them. The objective of the needs analysis here was to find out the needs of the employees in order to derive suitable interventions. Our main objective was therefore not to substantiate existing data, but to map individual needs of the organization. We have corrected the mistakenly reported employee numbers. Thank you for your comments!

# Reviewer report: Reviewer response for version 1

**DOI:** 10.21956/openreseurope.14262.r26601

**Published:** 2021-05-05

**Authors:** Michael Donnelly

**Affiliations:** 1 Centre for Public Health, Queen's University Belfast, Belfast, UK

This paper is described as presenting a qualitative study about perceptions regarding the important topic of mental health in workplace settings. I have noted below a number of observations and queries that need to be addressed. It is possible that many queries could be addressed by clarifying the forms of expression that are presented in the paper and increasing its English language fluency.

 The introduction to the paper provides a useful overview and rationale for the study. However, there is no review or reference to qualitative studies on workplace mental health needs, interventions or potential side effects. How does this qualitative study relate to other qualitative studies on this topic? In particular, what is the scope of qualitative studies in relation to the research question that the study addressed?

 In the methods section, please clarify further how employees were invited to participate in the qualitative study, how many by type of employee were invited, how many x type consented and how many x type were interviewed. What was the nature of the personal invitation? How did managers decide who to nominate to be interviewed? How many were nominated? How many agreed? Comment in this section or in the Discussion section about the extent to which the qualitative (non-probability) sample captured the range of characteristics in the employee workforce and that the review literature indicated were pertinent to the focus of this qualitative study. Please check numbers e.g. 6418 employees on page 4 and 6488 employees on page 5; ‘…34 of the 40 personally invited reported back…’ but only 33 interviews were conducted (page 5). What ‘guidelines’ guided the conduct of the interviews? Please clarify the meaning of the use of the term ‘qualitative survey’ and how it differs from qualitative interviews. What are the sociodemographic characteristics of the 12 managers who were interviewed and the participants of the focus groups (the abstract states 5 FGs and the paper states 3 FGs)?

 The data appears to be presented in terms of ‘counts’ or the number of participants who stated a given view or response rather than in terms of a qualitative analysis. Did you give consideration to usual qualitative study concepts such as data saturation, the derivation of themes and supporting illustrative quotations or were these features deemed to be irrelevant? What does this paper add to existing published research, particularly to qualitative studies on this topic? The results and subsequent discussion are interesting though largely confirmatory with respect to previous research reports. Perhaps, the concept of ‘side effects’ in this context is a relatively novel finding and deserves greater attention. It may be beneficial for journal readers to learn how the data that is presented in this paper ‘mixes’ with the larger longitudinal study (of which it is part). Indeed, is it worth considering waiting until more data or richer data becomes available? It is difficult to conduct research and relatively easy to criticise study design and methodology - I hope that you find these observations constructive and helpful. Best wishes with the rest of your study.

Is the study design appropriate and does the work have academic merit?

Partly

Is the work clearly and accurately presented and does it cite the current literature?

Partly

If applicable, is the statistical analysis and its interpretation appropriate?

Not applicable

Are all the source data underlying the results available to ensure full reproducibility?

Partly

Are the conclusions drawn adequately supported by the results?

Partly

Are sufficient details of methods and analysis provided to allow replication by others?

Partly

Reviewer Expertise:

Public health and health services research including the evaluation of mental health care interventions and services.

I confirm that I have read this submission and believe that I have an appropriate level of expertise to confirm that it is of an acceptable scientific standard, however I have significant reservations, as outlined above.

# Response

**Published:** 2024-09-03

**Authors:** Beate Muschalla

**Affiliations:** Technische Universität Braunschweig, Germany

Many thanks for this helpful advice. We have added a paragraph in the theory section on the current state of qualitative studies on workplace mental health promotion (line 129-162).

 In addition, the following explanation was added to the “research question” section, which sets our study project apart from others: “In contrast to previous qualitative studies on workplace health promotion, the focus here is on a public institution and examines the specific needs of managers and employees for health-promoting activities. Side effects have not yet been taken into account in previous research on workplace health promotion and are included here.” (line 221-225). We have specified the method selection based on your comments. 

 In the “Discussion” section, we have added a corresponding limitation regarding the missing random sample (line 476-483). We also carried out the creation of the interview guidelines. The term “qualitative survey” was misleading here and has been replaced by “interviews”. The sociodemographic characteristics of the participants are listed in the “Participants” section of the methods section (average age, gender, research or administration).

 We have also added a further table of socio-demographic data (Table 1). More sociodemographic characteristics were not collected for anonymization purposes. We have reconciled the contradictory numbers and adjusted them accordingly.

 We appreciate your comments. The evaluation was carried out according to the standardized procedure of a qualitative content analysis, so that the interview transcripts were analyzed and categories were formed on an inductive basis. We have now added direct example quotes to the results section. Table 2 is a condensed presentation of the results based on the very large database, which would otherwise not be possible to depict. In the discussion section, we have explained in more detail what our research adds to the current study situation and have placed even more emphasis on side effects (line 413-417; line 470-473).

 As the study was conducted three years ago due to the extended review process, there is unfortunately no data available to include more data. A longitudinal format was not planned for the needs analysis as part of the project.
